# Supplementary material for: Pectenovarin, A New Ovarian Carotenoprotein from Japanese Scallop Mizuhopecten yessoensis
Source: Molecules. 2020 Jul 3;25(13):3042. doi: 10.3390/molecules25133042 (PMC7411991; doi:10.3390/molecules25133042)
Supplement: Supplementary file 1 [file molecules-25-03042-s001.pdf]

Supplementary Materials

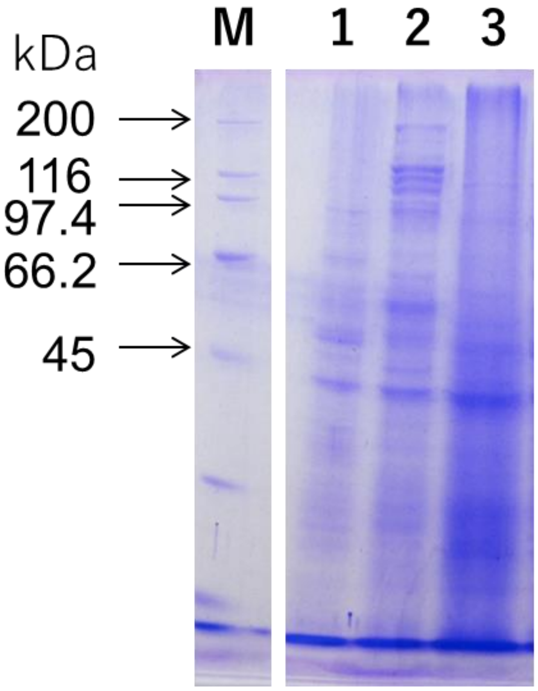

Figure S1. A comparison of crude extracts of the testis, ovary, and midgut gland

Freeze-dried specimen was extracted with the buffer to give extract (50 mg/mL), which was centrifuged at 12000 rpm, 4°C for 5 min. The 2 μL of supernatants was loaded to 10 % acrylamide gel and then stained with CBB.

M: size marker, 1: testis, 2: ovary, 3: midgut gland

A

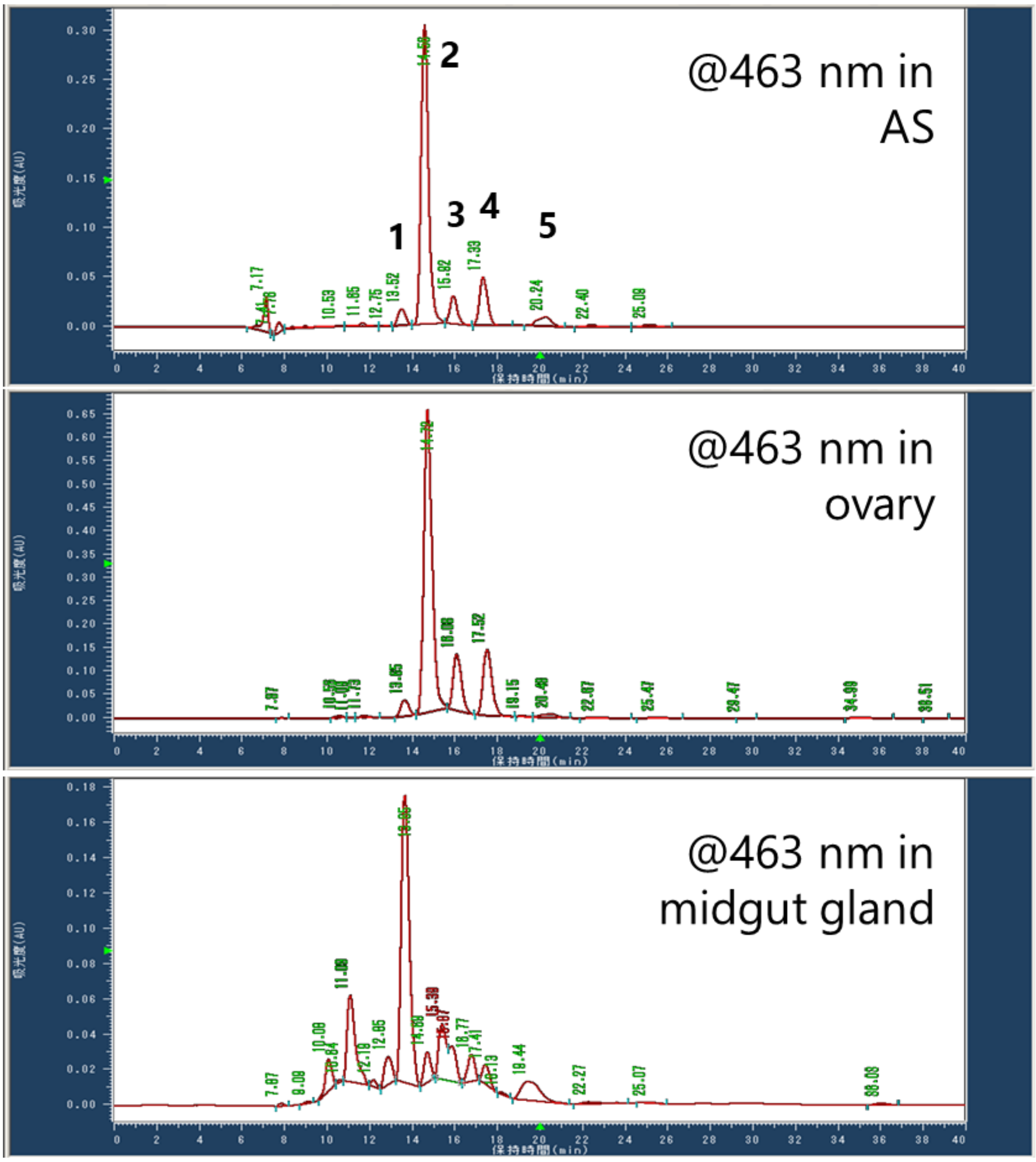

Figure S2A. PDA Chromatograms of carotenoid extracts from the freeze-dried scallop tissues at 463 nm. AS is ammonium sulfate precipitation of the ovary.

B

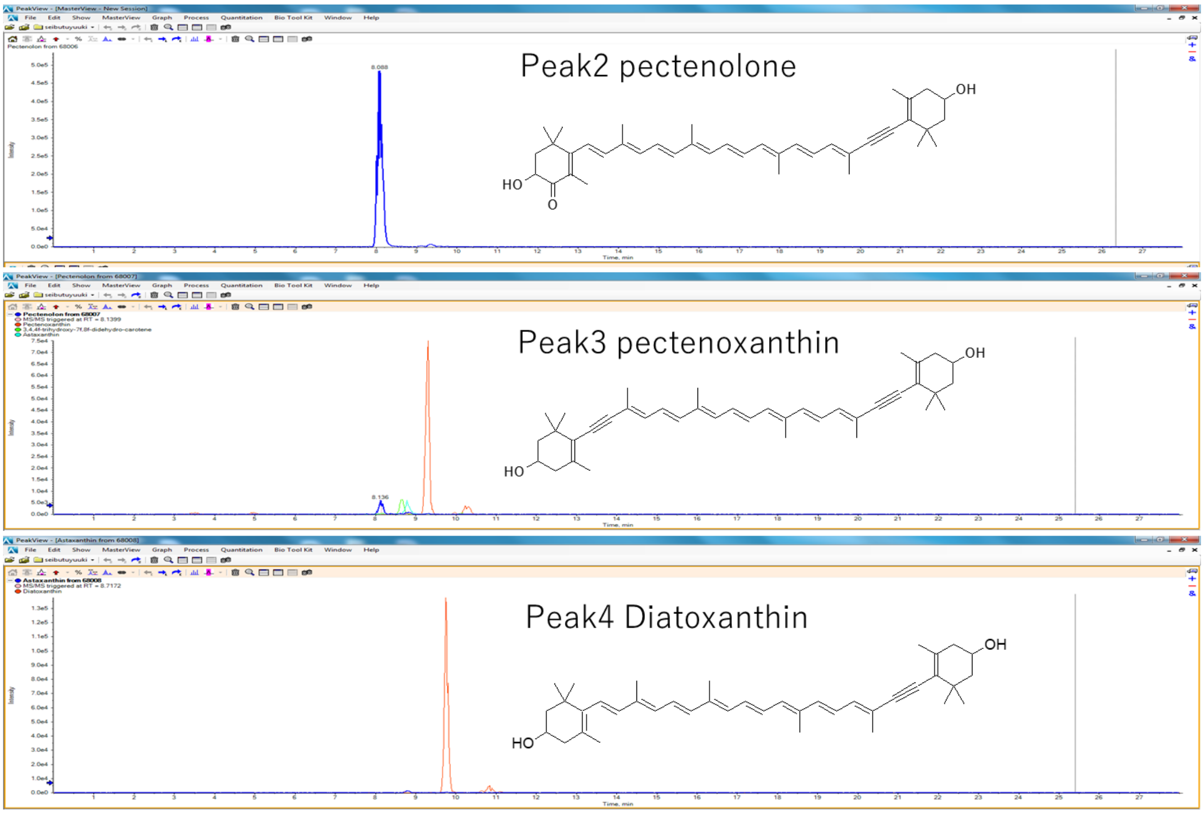

Figure S2B. **Mass chromatograms for Peak 2-4 (see, Figure S2A) analyzed by HRESI-MS/MS.**

Table S1.

The distribution of short fragments obtained by De Novo sequencing analysis on the deductive aminoacid sequence of the vitellogenin of *M. yessoensis*

The first amino acids and the last one of the distribution are exhibited on the table. The number from one to five correspond to the bands on electrophoresis in Figure 1F.

	simirality %	First Residue no.	AA	Last Residue no.	AA
1	92.7	80	I	1149	D
2	78.8	956	A	1660	K
3	86.3	588	S	2166	Q
4	87.4	32	Y	1888	R
5	81.3	80	I	1888	R
